# Supplementary material for: Expression of Hairpin-Enriched Mitochondrial DNA in Two Hairworm Species (Nematomorpha)
Source: Int J Mol Sci. 2023 Jul 13;24(14):11411. doi: 10.3390/ijms241411411 (PMC10380579; doi:10.3390/ijms241411411)
Supplement: Supplementary file 1 [file ijms-24-11411-s001.zip › Figure_S1.pdf]

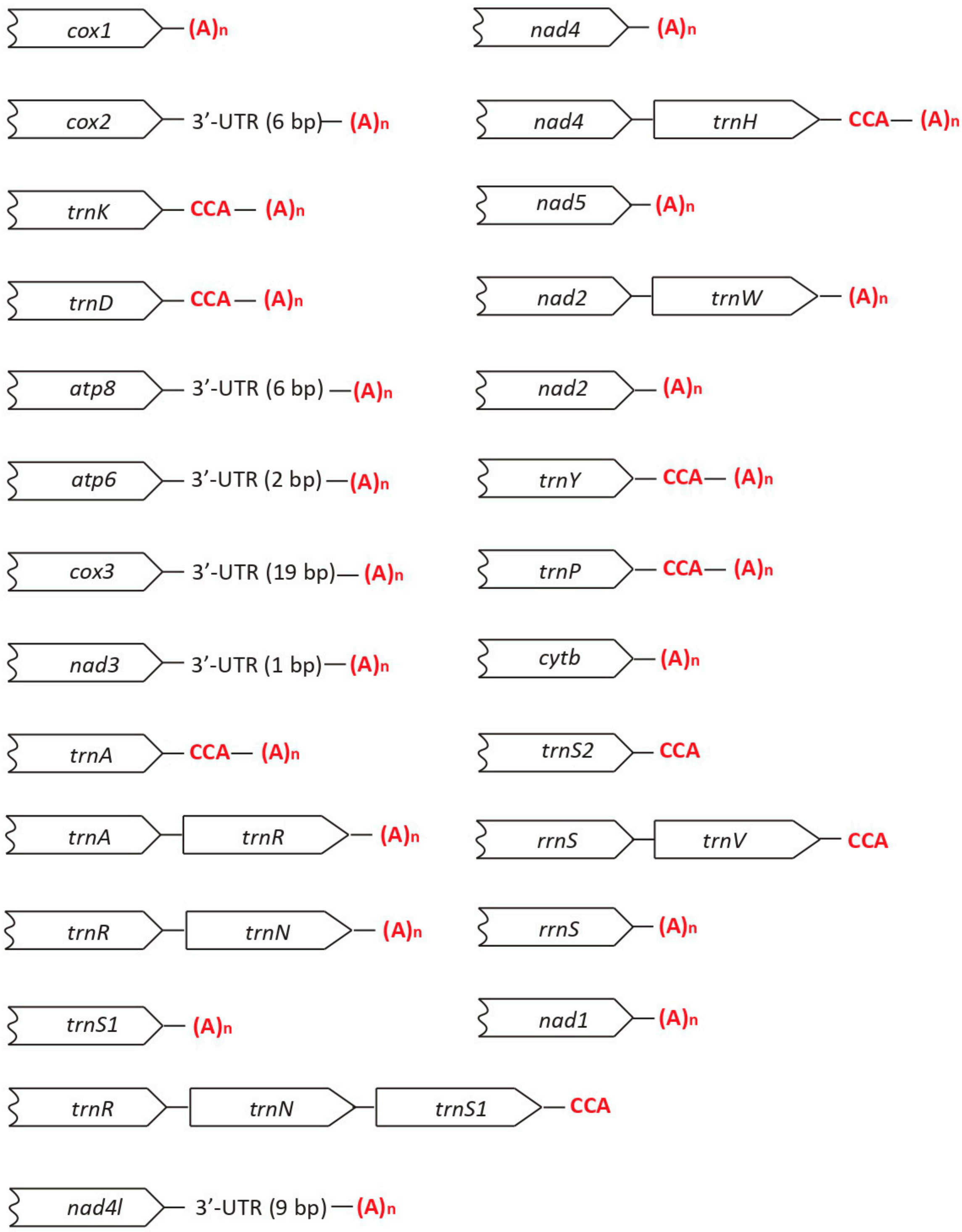

Figure S1. Transcripts modifications according to RNA-seq. Modifications are colored with red. We manually analyzed RNA-seq coverage of *Parachordodes pustulosus* mitochondrial genome and found some RNA read modifications. Unidentified 5′ ends of transcripts are jagged. The lengths of 3′ UTRs after some PCGs are given in brackets. Polyadenylation is signed with $(A)_n$. Non-template adding of nucleotides (CCA) afterwards tRNA-precursors are signed with CCA. At least some tRNA-precursors are modified with CCA and polyadenylated afterwards during the maturation process.
