# Supplementary material for: Expression of Hairpin-Enriched Mitochondrial DNA in Two Hairworm Species (Nematomorpha)
Source: Int J Mol Sci. 2023 Jul 13;24(14):11411. doi: 10.3390/ijms241411411 (PMC10380579; doi:10.3390/ijms241411411)
Supplement: Supplementary file 1 [file ijms-24-11411-s001.zip › Figure_S2.pdf]

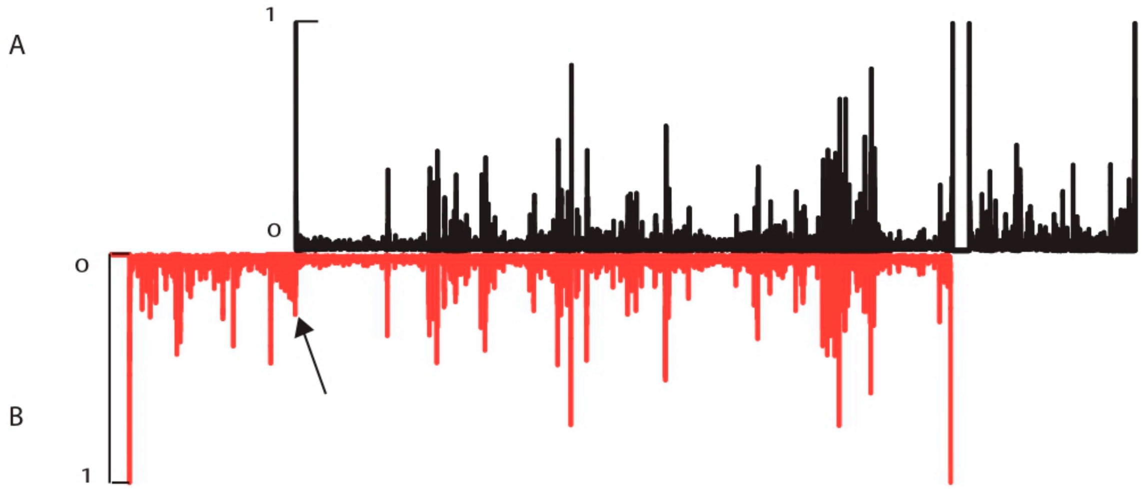

Figure S2. The ratio of 5'+3' ends to the total coverage of RNA-reads. (**A**). Mitochondrial genome starting with *cox1*. (**B**). Mitochondrial genome starting with the 10,947 site. The arrow points to the beginning of *cox1* gene.
